# Field Method for Testing Repellency of an Icaridin-Containing Skin Lotion against Vespid Wasps

**DOI:** 10.3390/insects7020022

**Published:** 2016-06-03

**Authors:** Jean-Luc Boevé, Frank Eertmans, Els Adriaens, Bart Rossel

**Affiliations:** 1 Service Entomology, O.D. Taxonomy and Phylogeny, Royal Belgian Institute of Natural Sciences, Rue Vautier 29, Brussels B-1000, Belgium; 2 Oystershell Laboratories, Booiebos 24, Drongen B-9031, Belgium; frank.eertmans@oystershell.be (F.E.); bart.rossel@oystershell.com (B.R.); 3 Bellemdorpweg 95, Aalter B-9981, Belgium; adriaens.consulting@telenet.be

**Keywords:** vespids, *Vespula vulgaris*, field tests, body cream, repellency, icaridin

## Abstract

Vespid wasps are ecologically beneficial predators of insects but their stings also pose a human health risk. Current control methods based on killing vespids are suboptimal. Here, the repellent effect against *Vespula vulgaris* of a 20% icaridin skin lotion was evaluated under field conditions. An experimental setup was designed in which six artificial skin pieces (10 × 10 cm) were video-recorded for 1 h, to count each min the numbers of flying and feeding vespids. Prior to monitoring, five pieces were successively smeared with 2 mg of cream per cm^2^, in 30 min intervals, from *t* = −120 min to 0. The sixth sheet remained untreated to serve as a control. One milliliter of an attractant, fruit jam, was deposited on each of the six surfaces at *t* = 0. The control surface was free of any flying or feeding vespid during an average period of 25 min, whereas the other five surfaces (treated at *t* = −120, −90, −60, −30, and 0 min) remained vespid-free for 39, 40, 45, 49, and 51 min, respectively. The skin lotion remained significantly active for at least 2 h. The experimental methodology is adjustable and allows the study of repellents against vespids in semi-natural conditions.

## 1. Introduction

Vespid wasps (Hymenoptera, Vespidae) are beneficial in regulating terrestrial ecosystems since they are significant predators of other insects. This significance is sustained by their abundance, itself due to their social behavior and, for some species, their tendency to become invasive and opportunist. However, besides prey items and other protein sources, they are also highly attracted by carbohydrates. They often forage around food sources and garbage in places where people are relaxing (e.g., recreational parks) or working (in forestry, arboriculture, horticulture, *etc.*). Since vespids defend themselves and their colony by stinging, they constitute a nuisance and even a serious hazard [1,2].

Consequently, applied research aims to find control methods to limit or reduce negative interactions between people and vespid populations. The destruction of nests is clearly a drastic method and it can indirectly increase pest populations. The alternative use of toxic baits, while less devastating to the vespids, are often harmful to beneficial arthropods [3]. Control by baiting may be considered as a lesser evil, but it works only if people stay in the neighborhood of the trap. Recent studies of several essential oils and single volatiles demonstrate their repellent effects against vespids [4,5], but some volatiles can attract vespids [6,7]. Such chemicals may be integrated in a “push-pull” strategy that combines repellents around people, and attractants at some distance from them, to trap the vespids [8]. This method remains, however, unsatisfactory because it still kills the insects. As an alternative, essential oils-based repellent products (*i.e.*, portable hanging diffuser and reusable decorative shell) are commercially available in North America.

Here, we describe an experimental setup, to assess the repellent effect of a skin lotion, containing 20% icaridin, against vespids under field conditions. To the best of our knowledge, such a field bioassay tool is new. For safety and practical reasons, no human volunteers were involved; hence, artificial skin sheets were used instead. Results are discussed by appraising pros and cons of the setup as well as the effectiveness of the lotion.

## 2. Materials and Methods

### 2.1. Insects

All tests were performed in the field near an apple orchard with vespid populations (Auderghem, Belgium) between 09:00 and 18:00, from 10 to 21 August 2015. If necessary, a sugared liquid such as grenadine syrup was left at the test place overnight and/or during testing, to maintain a sufficient amount of vespids present in the study area.

Throughout the test period, 43 vespids were randomly collected for later identification. All these specimens belong to the species *Vespula vulgaris* (Linnaeus). This species and *Vespula germanica* (Fabricius) are the most common vespids in Europe.

### 2.2. Material

An experiment was designed to evaluate the repellency of Picasol^®^ Aftersun and Insect Protection (Oystershell Laboratories, Drongen, Belgium), a skin lotion with a proprietary formulation (20% icaridin) to protect against vespid wasps as well as other insects and ticks.

The substrate on which the product was smeared consisted of a 10 cm × 10 cm piece of an artificial skin (PFT Sheets of Tattoo Practice Skin; purchased on Amazon; thickness: *ca.* 1 mm). Before use, these artificial skins were soaked in soapy water for at least 24 h, and then rinsed and soaked in water for at least 24 h. The amount of the product corresponded to an equivalent of 2 mg/cm^2^. Thus, 220 µL of the skin lotion were deposited on the skin piece by using a BRAND Transferpettor (100–500 µL) and smeared as evenly as possible on that surface, using the rounded tip (diameter: 1 cm) of a glass cylinder. The skin pieces were placed on a paper sheet (A3 format) sitting on a hard plate on the ground. The attractant used in the test was a four-berry jam (total sugar content: 60 g per 100 g) that was sieved before use to eliminate most pulp particles. Video filming was made with a Panasonic HC-VX870 camera, mounted vertically to film the paper sheet from above. The paper sheet, skin pieces, skin lotion, and attractant were renewed between each replication.

### 2.3. Field Experiments

**Figure 1:**
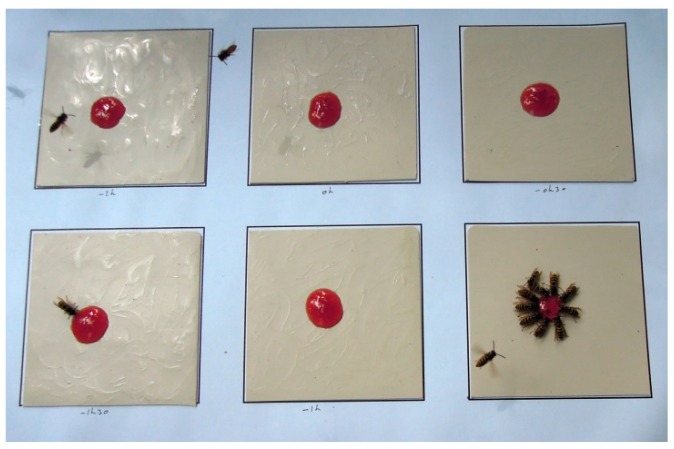
Picture of the running field test that involved *Vespula vulgaris*. Jam was used as an attractant. In the example shown here, the only surface not treated with the skin lotion was the one on the lower right corner. For further explanation, see text.

For each replicate, six skin pieces were placed on an A3 paper sheet (landscape orientation), in two horizontal rows of three and spaced out with a margin of 2.5 cm. The video sequence started at *t* = 0 and lasted 60 min. The skin lotion was smeared once and at random on one of the skin pieces at *t* = −120 min, −90 min, −60 min, −30 min, and at *t* = 0; this left one skin piece untreated as control. Then, 1 mL attractant was deposited in the center on each skin surface (at *t* = 0). Thus, the video sequence documented the repellent activity of the skin lotion, relative to the control surface (Figure 1), 0 to 3 h after deposition of that product. The ambient temperature (0.5 °C precision) was recorded each 30 min from *t* = −2 h to +1 h. In total, the test was repeated 18 times, totalizing 90 skin lotion applications.

### 2.4. Data Analysis

Video clips were analyzed every minute of the 1 h observation period to determine the number of vespids feeding on each spot of the attractant as well as the number of individuals flying a few centimeters over each skin piece. The data were also expressed as the total vespid-free time (*i.e.*, no flying or feeding vespid), to avoid calculation biases due to temporal auto-replicated data. The mean vespid-free time for the different treatments was calculated using a one-way analysis of variance (one-way ANOVA). The normality of the residuals was investigated with a qq-plot. Homogeneity of variances was tested with the Levene’s test. In case of a significant ANOVA, Tukey’s procedure was used to establish the efficacy of the treated *vs.* control skin sheets. A value of *p* < 0.05 was considered statistically significant. All statistical analyses were performed in R version 3.2.0 [9].

The time needed for the vespids to consume the attractant was also determined from the video files.

### 2.5. Skin Lotion Evaporation

Evaporation of the skin lotion from the artificial sheet was assessed under laboratory conditions by immediately placing a treated surface on an analytical balance (0.1 mg precision) and measuring the weight in 5 to 30 min-intervals over 3 h, at 20 °C and 25 °C (three replications per temperature).

## 3. Results

**Figure 2:**
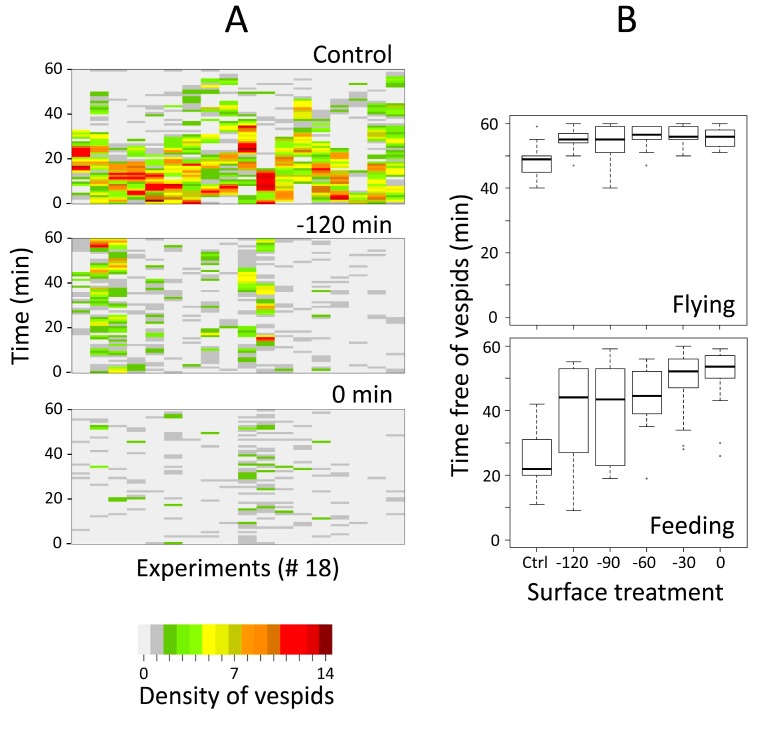
Skin lotion repellency against *Vespula vulgaris* tested under field conditions. (**A**) Heat-maps of the total numbers of vespids in function of time, for three surfaces (control, treated at *t* = −120 min and *t* = 0). The X-axis represents the replicated experiments, the Y-axis the number of vespids counted at each min, following the colored scale bar (*below*). (**B**) Box-and-whisker plots of the overall numbers of time counts without flying and feeding vespids, for each one of the six surfaces: (Ctrl) control, (−120 to 0) min.

More vespids counted on the six artificial skins were feeding (average: 69.9%) than flying (30.1%). More vespids were flying or feeding on the control surface than on the surfaces treated 120 to 0 min prior to video recording (Figure 2A). The Pearson’s correlation coefficients between the number of vespids observed on the control surface compared to the treated surfaces were 0.09, 0.33, 0.33, 0.23, and 0.40 for the surfaces treated at *t* = −120, −90, −60, −30, and 0 min, respectively.

The attractant was completely consumed within 60 min for 12 out of 18 control surfaces, and 1 (treated at −90 min) out of 90 treated surfaces (*p* < 0.001, Fisher exact probability test, two-tailed; *n* = 108). As the attractant on the control surface was consumed, the vespids gradually switched to the other surfaces, preferring the earliest treated ones (at *t* = −120 and −90 min; Table 1).

All treated sheets compared to the control yielded significantly longer vespid-free times (Figure 2B, Table 1). The mean (and median) vespid-free time was 39.2 (44.0), 39.9 (43.5), 44.7 (44.5), 49.4 (52.0), and 50.6 (53.5) min for sheets treated 120, 90, 60, 30, and 0 min prior to video recording, respectively; the control surface remained vespid-free during 24.7 (22.0) min. These data are illustrated in Figure 2B, specifically for times free of flying and feeding vespids.

During the field testing, average temperature per experiment ranged from 20.5 °C to 25.5 °C. In the laboratory, the weight (expressing the evaporation rate) of the skin lotion decreased exponentially, showing a 50% decrease after 15 min at 20 °C, and 10 min at 25 °C. At both temperatures, 45%–46% of the initial weight remained after 2 h and also 3 h.

## 4. Discussion

To test a repellent activity, as for any bioactivity, it is essential to adapt the experimental setup in line with the target organism. Repellent or biocidal compounds are often formulated in a spray or body skin lotion to act against harmful arthropod species which mainly belong to the taxa Diptera and Acari [10,11]. To the best of our knowledge, no publication describes the testing of a skin lotion against wasps in the field. Under the experimental conditions reported here, such a product was able to repel vespids for at least 2 h, which is typically a minimum protection time required for commercial registration (e.g., [12]). From a more general point of view, the multiple choice setup allowed evaluation of the repellency of the icaridin-based skin lotion, applied at different times, but evaluation occurring in identical spatiotemporal conditions of field temperature, wasp densities, *etc.*

However, we recognize that the setup has some limitations. First, the whole filmed experimental zone (*i.e.*, paper sheet plus artificial skins) may become saturated with a “repellent cloud”, preventing vespids from flying around this area and from eventually detecting the spots of attractant. The solution of spreading the test sheets further apart may however reduce the video quality, but it would probably increase the significance level of the statistical results.

Second, only the species *V. vulgaris* was present on the testing site. While we expect that *V. germanica* and other *Vespula* species would be repelled in a similar fashion, the repellency against species from the genera *Dolichovespula*, *Vespa*, and *Polistes* needs to be confirmed.

Third, the skin lotion may have slowly impregnated the spot of jam used as attractant, since the latter was deposited on the former for convenience. The skin lotion lost part of its repellency over time (Figure 2B), but bioactivity was measured relative to an attraction source (*i.e.*, the jam), which is the usual method of testing repellency. Moreover, there is also no clear-cut limit between repellency (by olfaction) and deterrence (gustation) caused by “volatile” compounds [10,13]. Hence, it is unlikely that the mix of skin lotion and jam confounded the results.

## 5. Conclusions

Our observations suggest that the 20% icaridin skin lotion repels vespids at a short range as flying individuals were significantly more abundant on the control surface than on any of the treated surfaces (Figure 2B). Further, the present experiment demonstrates that the 20% icaridin skin lotion repelled vespids for at least 2 h, hereby lowering the risk of being stung. Interestingly, the experimental setup may be adapted to study the repellency of any volatiles, mixed or not. Possible practical adaptations may include: (1) using different compounds on each test surface instead of studying the effect of time elapsed since product application; (2) spacing this time more than by steps of 30 min; and/or (3) delivering chemical(s) in such a way that no contact is made with the attractant.

## Figures and Tables

**Table 1:** Statistical comparisons between vespid-free times on an untreated control surface and on five surfaces treated at different times.

Treatment Comparisons	Mean Difference	Lower 95% CI	Upper 95% CI	*p*-adj
Control *vs.* −120 min	14.4	3.3	25.5	0.004
Control *vs.* −90 min	15.2	4.1	26.3	0.002
Control *vs.* −60 min	20.0	8.9	31.1	<0.001
Control *vs.* −30 min	24.7	13.6	35.8	<0.001
Control *vs.* 0 min	25.9	14.8	37.0	<0.001
−120 *vs.* 0 min	11.4	0.3	22.5	0.039

Mean differences and confidence intervals (CI) are mentioned in min. (*p*-adj) Adjusted *p* values, which correspond to statistically significant differences between group means as determined by one-way ANOVA: *F*_5,120_ = 12.18, *p* < 0.001, Tukey post-hoc multiple comparison of means.
